# Draft Genome Sequences of *Klebsiella* Species Isolated from the International Space Station

**DOI:** 10.1128/MRA.00923-20

**Published:** 2020-10-15

**Authors:** Samuel A. Solomon, Achintya R. Bharadwaj, Nitin K. Singh, Jason M. Wood, Marilyne Debieu, Niamh B. O’Hara, Christopher E. Mason, Kasthuri Venkateswaran

**Affiliations:** a Jet Propulsion Laboratory, California Institute of Technology, Pasadena, California, USA; b Biotia, New York, New York, USA; c Department of Cell Biology, College of Medicine, SUNY Downstate Health Sciences University, Brooklyn, New York, USA; d WorldQuant Initiative for Quantitative Prediction, Weill Cornell Medicine, New York, New York, USA; e Department of Physiology and Biophysics, Weill Cornell Medicine, New York, New York, USA; University of Arizona

## Abstract

Isolated across four locations aboard the International Space Station (ISS), 10 bacterial strains were compared using whole-genome sequencing analysis and were phylogenetically identified as *Klebsiella*. The whole-genome sequences will aid in comparative genomic studies of ISS *Klebsiella* strains with Earth counterparts to gain insight into their adaptation to space conditions.

## Abstract (precis)

Isolated across four locations aboard the International Space Station (ISS), 10 bacterial strains were compared using whole-genome sequencing analysis and were phylogenetically identified as *Klebsiella*. The whole-genome sequences will aid in comparative genomic studies of ISS *Klebsiella* strains with Earth counterparts, to gain insight into their adaptation to space conditions.

## ANNOUNCEMENT

The genus *Klebsiella* was discovered by Carl Friedlander in 1882 from the lungs of patients who had died of pneumonia (1). In infected individuals, *Klebsiella* species can populate the gastrointestinal tract and nasopharynx, surviving on mucosal surfaces, and are known for being highly virulent and resistant to antibiotics (2, 3). When found on Earth, this genus of bacterial pathogens has various degrees of pathogenicity, which can lead to severe breathing problems necessitating a ventilator and rapid on-site treatment (4). When exposed to space conditions, however, *Klebsiella* species might become immunogenic and thus pose a risk to already immunocompromised astronauts aboard the International Space Station (ISS) (5, 6). Since microgravity and radiation in space are reported to induce multiple genetic adaptations in microbial species, such as structural modifications of the cell membrane, that can subsequently alter their virulence (5), the strains identified here might potentially pose a problem for the health of astronauts. Therefore, a genetic comparison is necessary to provide more details on the survival of *Klebsiella* species, which have gained more attention because classical Klebsiella pneumoniae and its hypervirulent pathotype are becoming increasingly resistant to various antibiotics, such as carbapenems (3, 4, 7). The pathogenicity and resistance of the members of the genus *Klebsiella* might potentially create a problem for space travel, specifically for the safety of astronauts.

Several strains of *Klebsiella* species, including K. aerogenes (*n* = 1), K. pneumoniae (*n* = 1), and K. quasipneumoniae (*n* = 8), were isolated from various locations on ISS environmental surfaces (8). The flight number, location, and other sampling characteristics of the ISS *Klebsiella* isolates are detailed in Table 1. Briefly, the environmental samples collected from the ISS and subsequently brought down to Earth at room temperature were aseptically handled according to established procedures (8), and 100-μl aliquots of concentrated samples were spread onto either Reasoner’s 2A (R2A) agar (25°C for 7 days) or blood agar (37°C for 2 days) for isolation of microorganisms. After morphological observation, pure colonies were archived at −80°C until further analyses. Cultures of the 10 *Klebsiella* strains were grown overnight on tryptic soy agar at 25°C until harvesting and DNA extraction using the ZymoBIOMICS DNA Magbead kit.

**TABLE 1:** Metadata and genome statistics of *Klebsiella* strains isolated from various ISS environmental surfaces during the Microbial Tracking-1 flight project

Sample name	Nearest species^*a*^	ANI (%)^*b*^	GenBank accession no.	Raw read accession no.	Flight-location^*c*^	Location description^*d*^	No. of contigs	Genome size (bp)	*N*_50_ (bp)	Median coverage (×)	No. of raw reads (×10^6^)	No. of quality-controlled reads (×10^6^)
IIIF7SW-P1	K. aerogenes ATCC 13048^T^	98.66	JACBPC000000000	SRR12071884	F3-7	Lab 3	25	5,155,046	502,580	346.88	21.6	21.5
F3-2P(2*)	K. pneumoniae ATCC 13883^T^	99.01	JACAUF000000000	SRR12068826	F3-2	WHC	75	5,489,009	332,420	293.62	19.0	18.9
IF1SW-B2	K. quasipneumoniae 01A030^T^	96.54	JABWPD000000000	SRR11884995	F1-1	Cupola	24	5,192,853	601,624	533.04	31.4	31.2
IF1SW-P3	K. quasipneumoniae 01A030^T^	96.60	JABWOZ000000000	SRR11885008	F1-1	Cupola	28	5,192,422	600,611	467.41	28.2	28.0
IF1SW-P4	K. quasipneumoniae 01A030^T^	96.55	JABWPA000000000	SRR11885009	F1-1	Cupola	29	5,192,468	343,111	297.32	18.6	18.6
IF2SW-B3	K. quasipneumoniae 01A030^T^	96.54	JABWPC000000000	SRR11885011	F1-2	WHC	24	5,192,354	600,611	712.50	39.8	39.6
IF2SW-P1	K. quasipneumoniae 01A030^T^	96.61	JABWPB000000000	SRR11885010	F1-2	WHC	27	5,192,385	601,139	480.80	30.2	30.1
IIIF3SW-P1	K. quasipneumoniae 01A030^T^	96.63	JABXWM000000000	SRR12070037	F3-3	ARED	31	5,154,415	766,557	286.96	18.0	17.8
F3-6P(1)	K. quasipneumoniae 01A030^T^	96.57	JABXWL000000000	SRR12070038	F3-6	PMM	28	5,196,291	1,009,008	371.62	23.2	22.9
F3-6P(2)	K. quasipneumoniae 01A030^T^	96.60	JABXWK000000000	SRR12070039	F3-6	PMM	31	5,196,372	1,009,008	367.78	23.4	23.1

a The 16S rRNA gene sequences were retrieved from the whole-genome sequences of the queried genomes and subjected to BLAST analysis against type strains for all 16S rRNA sequences in the NCBI database. Bacterial species identity was determined when the queried sequence showed >97.5% similarity to the 16S rRNA gene sequence of the type strain. The whole-genome sequence of the nearest neighbor listed was selected for ANI evaluation.

b ANI calculations were carried out using the EZBioCloud ANI calculator (https://www.ezbiocloud.net/tools/ani) by comparing with the listed type strain.

c Hyphenated designations indicate the flight number followed by the location; for example, F3-7 indicates flight 3 and location 7.

d WHC, waste and hygiene compartment; ARED, advanced resistive exercise device; PMM, permanent multipurpose module port 1.

Genomes were sequenced using the Illumina (San Diego, CA) Nextera Flex protocol for library preparation, and a NovaSeq 6000 S4 flow cell (paired end, 2 × 150 bp) was used for paired-end sequencing. The quality was assessed with FastQC (v0.11.7) (9). Adapter trimming and quality filtering were then carried out with fastp (v0.20.0) (10). After quality control, the sequences were assembled using SPAdes (v3.11.1) (11). To assess the quality of the final sequences, a QUAST (v5.0.2) analysis (12) was performed to check the *N*_50_ values, the number of contigs, and the total genome length (Table 1). The GC contents are 54.96% for K. aerogenes, 57.25% for K. pneumoniae, and 58.11 to 58.13% for K. quasipneumoniae. The 16S rRNA gene sequences of the *Klebsiella* strains were compared to find the nearest neighbor, and phylogenic characterization was determined by calculating the average nucleotide identity (ANI) using the EZBioCloud calculator (13), in comparison with the respective type strains (K. aerogenes ATCC 13048^T^, K. pneumoniae ATCC 13883^T^, and K. quasipneumoniae 01A030^T^). Default parameters were used for all software.

### Data availability.

This whole-genome sequencing project has been deposited in GenBank, and the GenBank and raw read accession numbers are given in Table 1. The BioProject accession numbers are PRJNA635942, PRJNA640688, and PRJNA640693. Whole-genome sequencing data have also been deposited in NASA GeneLab (accession numbers GLDS-302, GLDS-309, and GLDS-311). The versions described in this paper are the first versions.
